# Supplementary material for: The classical pathway triggers pathogenic complement activation in membranous nephropathy
Source: Nat Commun. 2023 Jan 28;14:473. doi: 10.1038/s41467-023-36068-0 (PMC9884226; doi:10.1038/s41467-023-36068-0)
Supplement: Supplementary file 1 — Supplementary Information [file 41467_2023_36068_MOESM1_ESM.pdf]

Supplementary Appendix

for

The classical pathway triggers pathogenic complement activation in membranous nephropathy

by

Larissa Seifert, Gunther Zahner, Catherine Meyer-Schwesinger, Naemi Hickstein, Silke Dehde, Sonia Wulf, Sarah M.S. Köllner, Renke Lucas, Dominik Kylies, Sarah Froembling, Stephanie Zielinski, Oliver Kretz, Anna Borodovsky, Sergey Biniaminov, Yanyan Wang, Hong Cheng, Friedrich Koch-Nolte, Peter F. Zipfel, Helmut Hopfer, Victor G. Puelles, Ulf Panzer, Tobias B. Huber, Thorsten Wiech, and Nicola M. Tomas

Supplementary Fig. 1

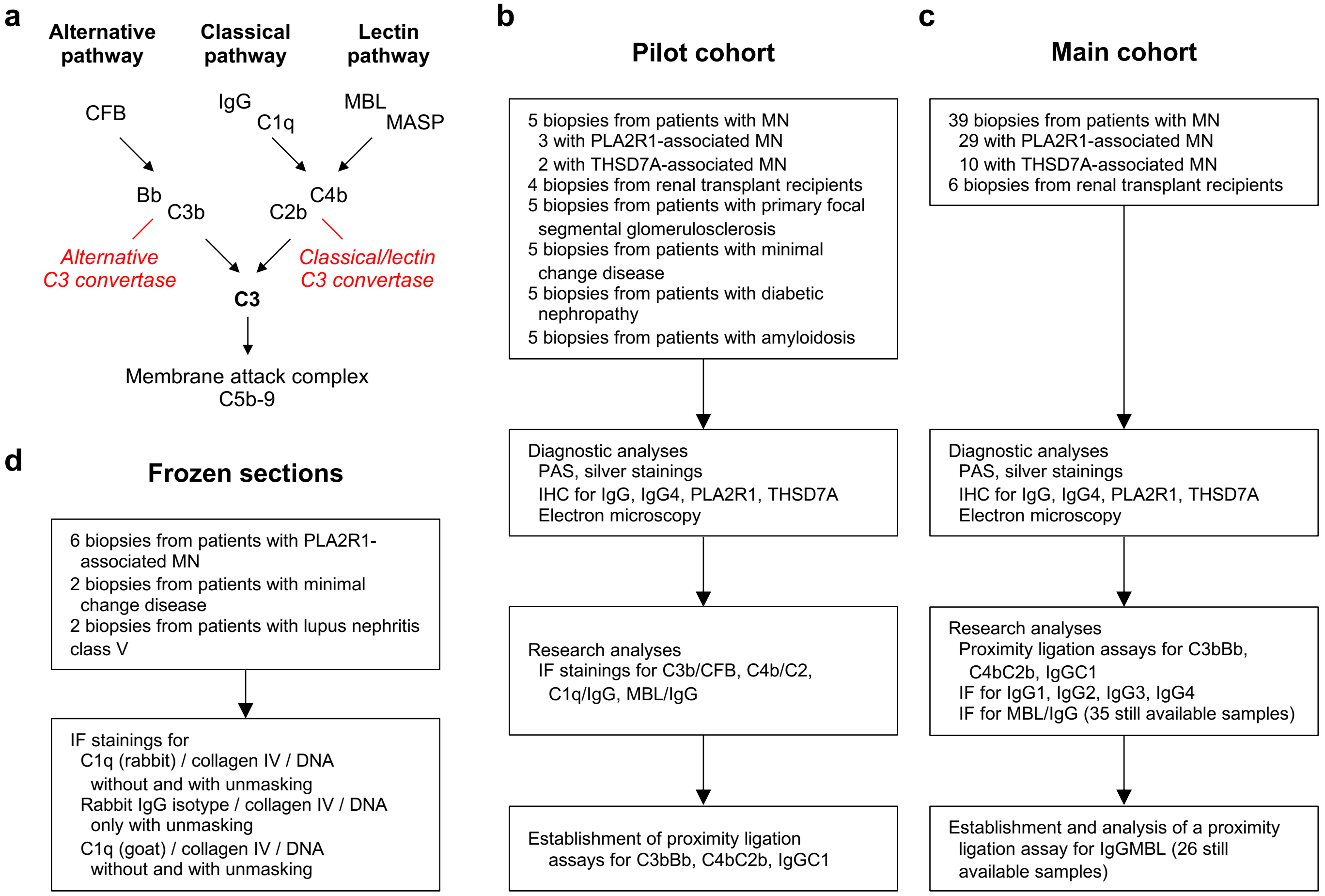

Supplementary Fig. 1 Complement cascade and retrospective study design. a Scheme of the complement cascade with its three activation pathways (alternative, classical and lectin) and involved key components. **b, c** Workflow of diagnostic and research analyses in the pilot (**b**) and the main cohort (**c**) of patients with PLA2R1- and THSD7A-associated MN as well as controls. (**d**) Stainings performed on frozen sections.
PAS, periodic-acid Schiff. IHC, immunohistochemistry. IF, immunofluorescence.

Supplementary Fig. 2

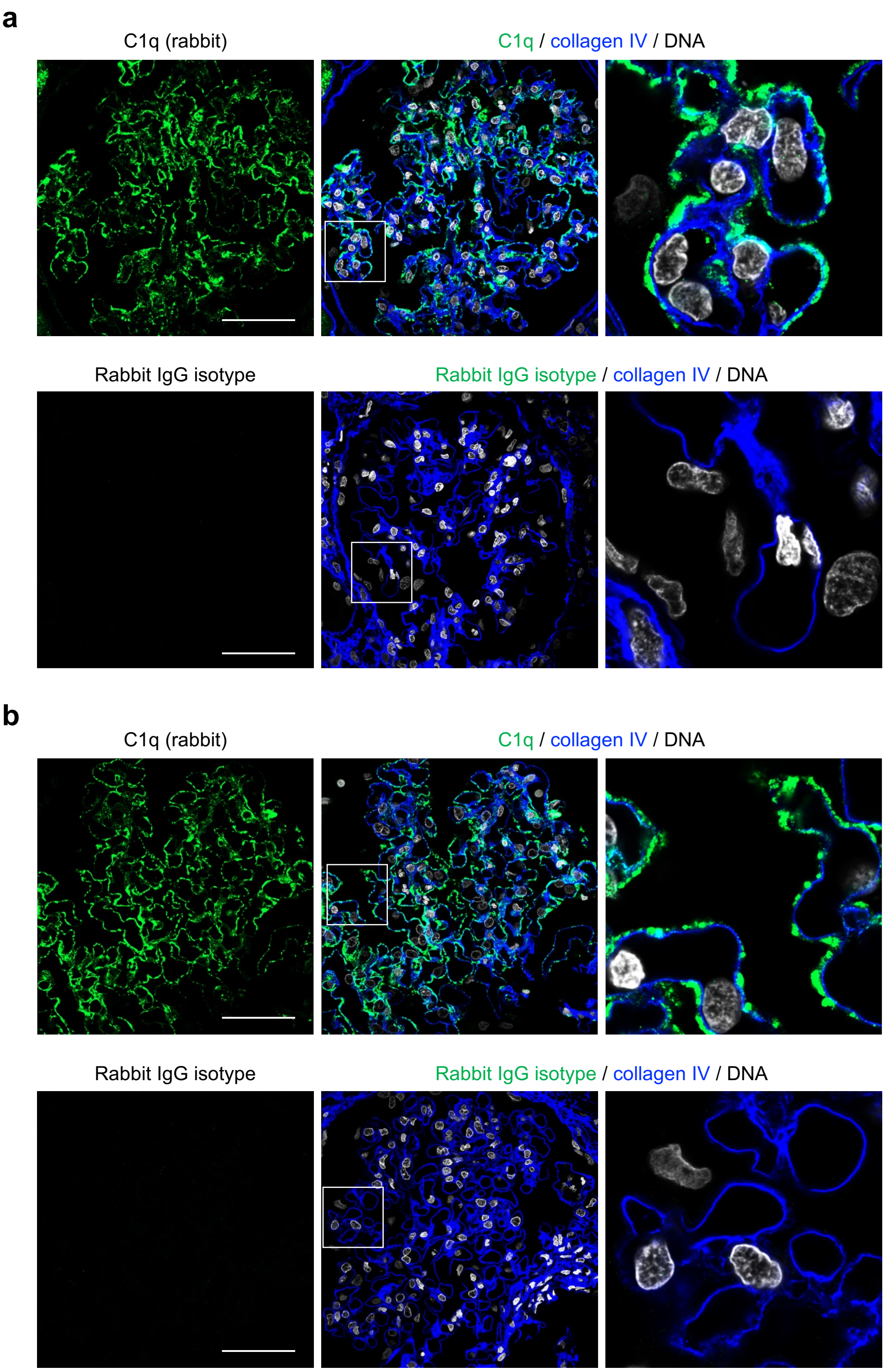

Supplementary Fig. 2 C1q positivity in biopsy samples (paraffin-embedded tissue) from MN patients is not a consequence of unspecific primary or secondary antibody binding. a, b Representative immunofluorescence stainings for C1q in co-localization with collagen IV in two cases (**a** and **b**) of PLA2R1-associated MN (upper panels). The same staining was performed replacing the primary rabbit anti-C1q antibody with an equally concentrated rabbit IgG isotype control (lower panels). A total of 5 biopsies were investigated like this with congruent results. The right panels represent 5-fold enlargements of the boxed areas in the middle panels. Bars 50 µm.

Supplementary Fig. 3

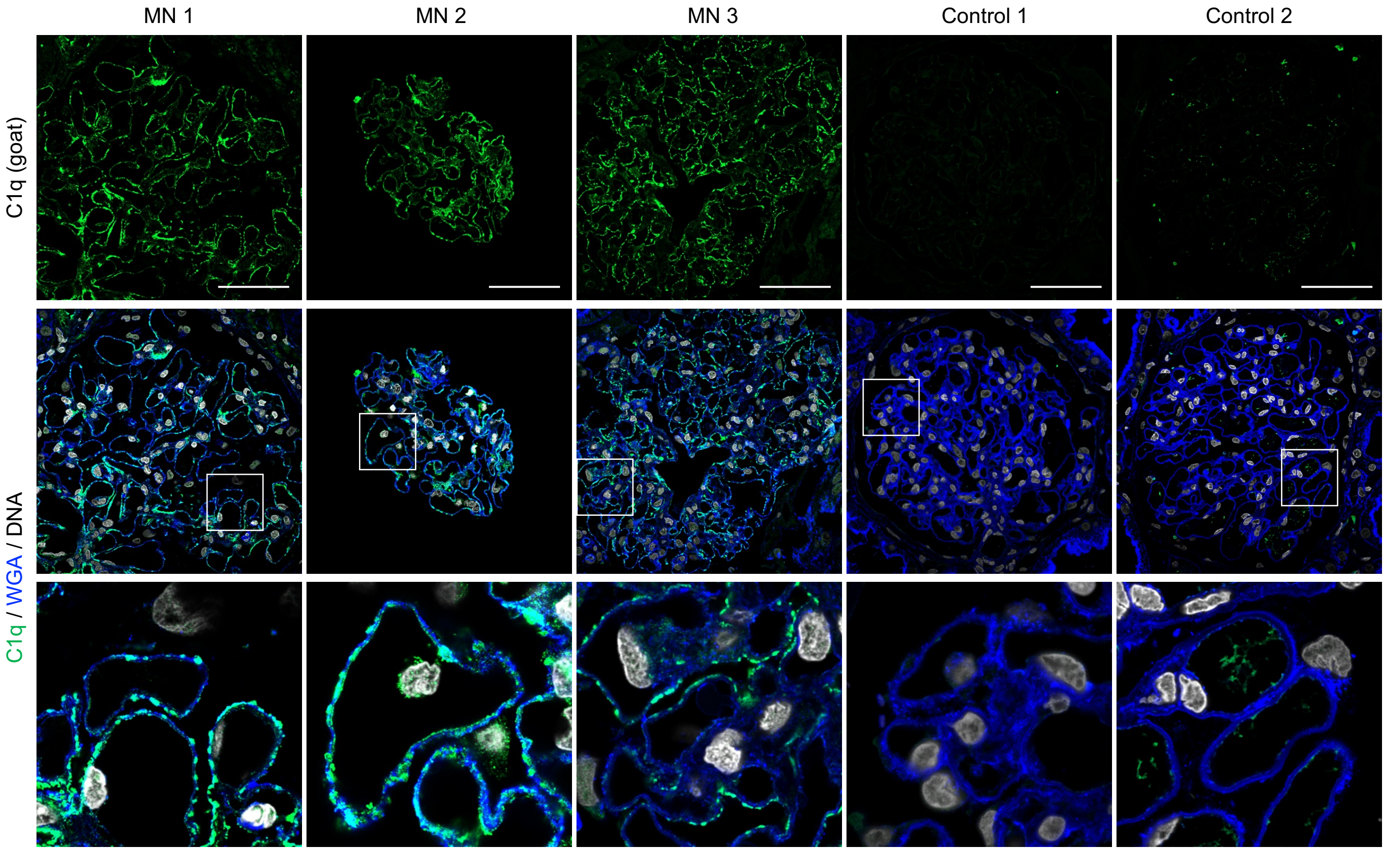

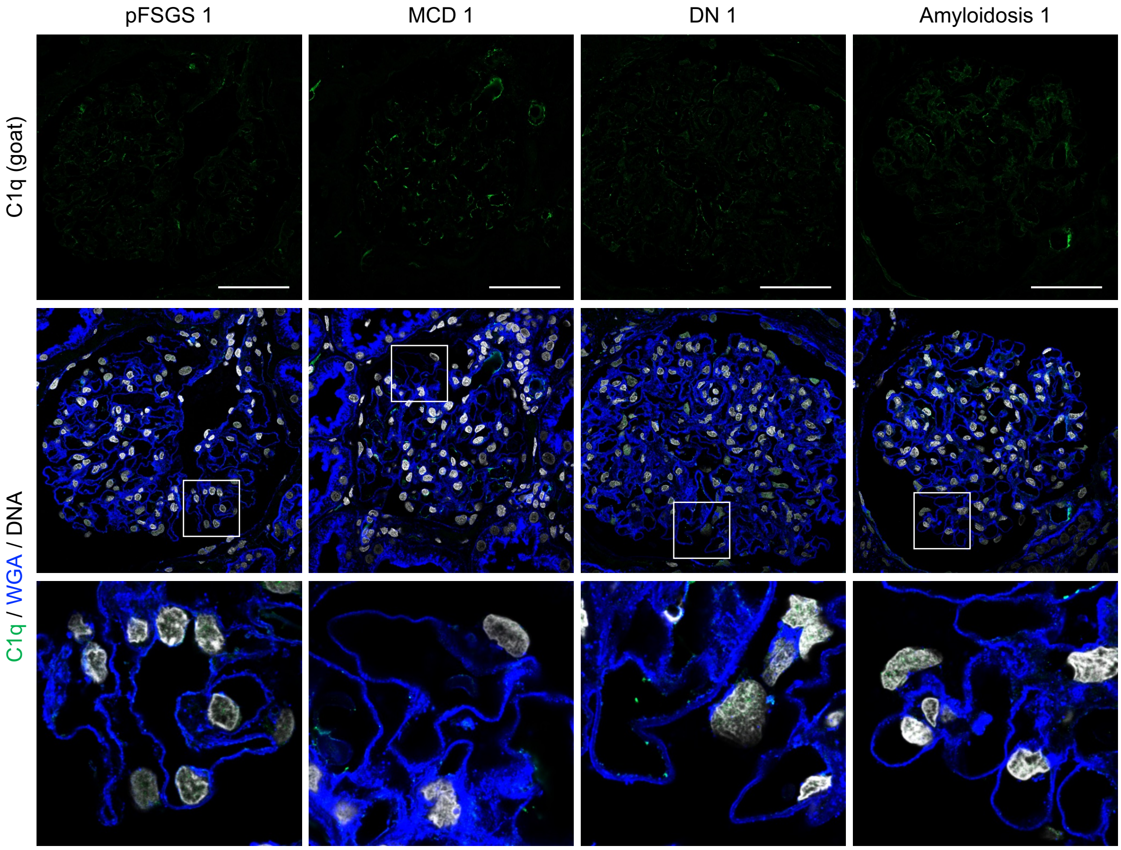

Supplementary Fig. 3 C1q positivity in biopsy samples from MN patients is confirmed with another anti-C1q antibody. Representative C1q stainings using a goat anti-C1q antibody in co-localization with wheat germ agglutinin (WGA) in three biopsies from patients with PLA2R1-associated MN (n=5 biopsies investigated in total), two time point zero biopsies from renal transplant recipients (control), and one biopsy each from patients with primary focal segmental glomerulosclerosis (pFSGS), minimal change disease (MCD), diabetic nephropathy (DN), and amyloidosis. For pFSGS, MCD, DN and amyloidosis, n=2 biopsies were analyzed in total. The lower panels represent 5-fold enlargements of the boxed areas in the middle panels. Bars 50 µm.

Supplementary Fig. 4

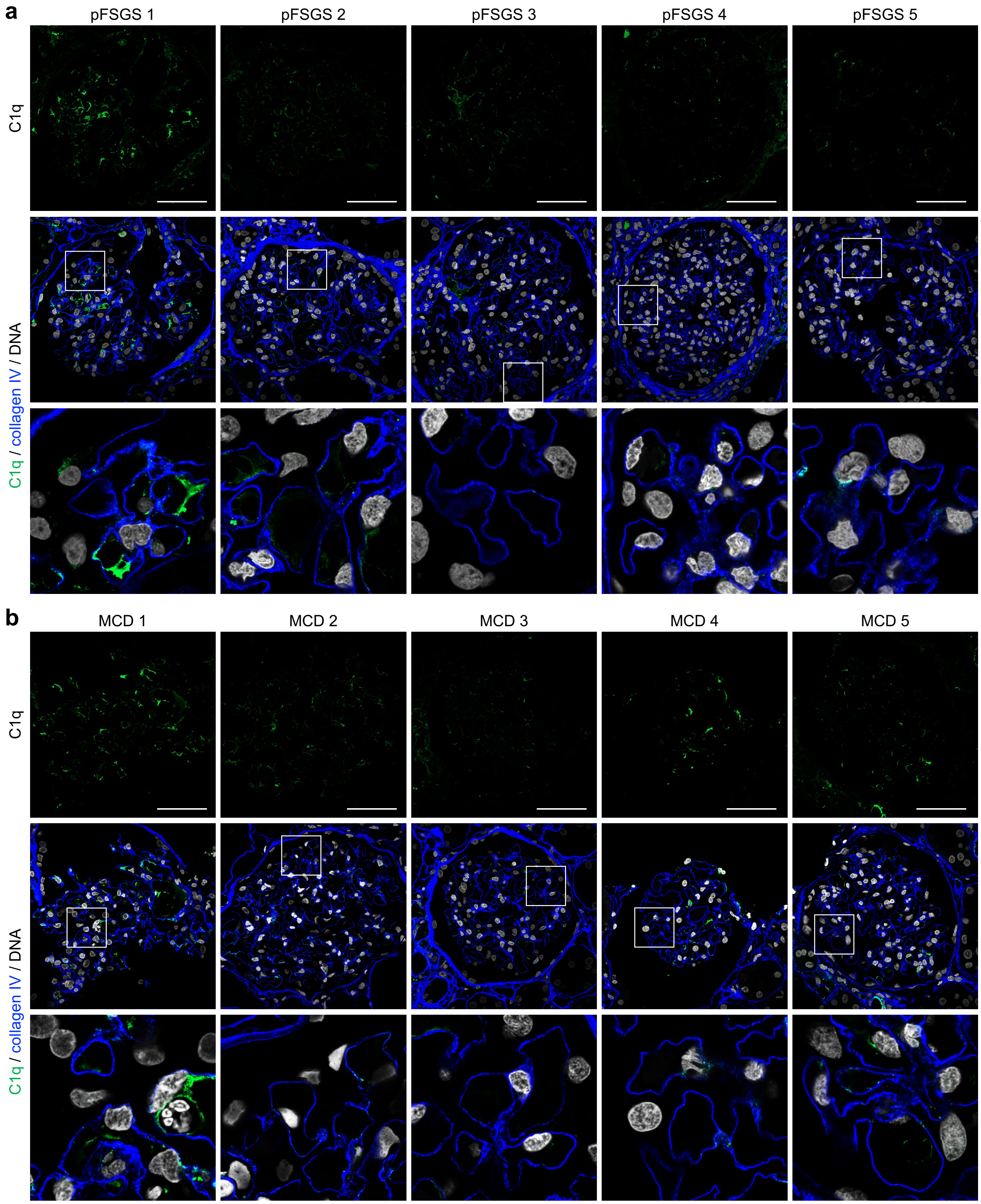

Supplementary Fig. 4 *(continued)*

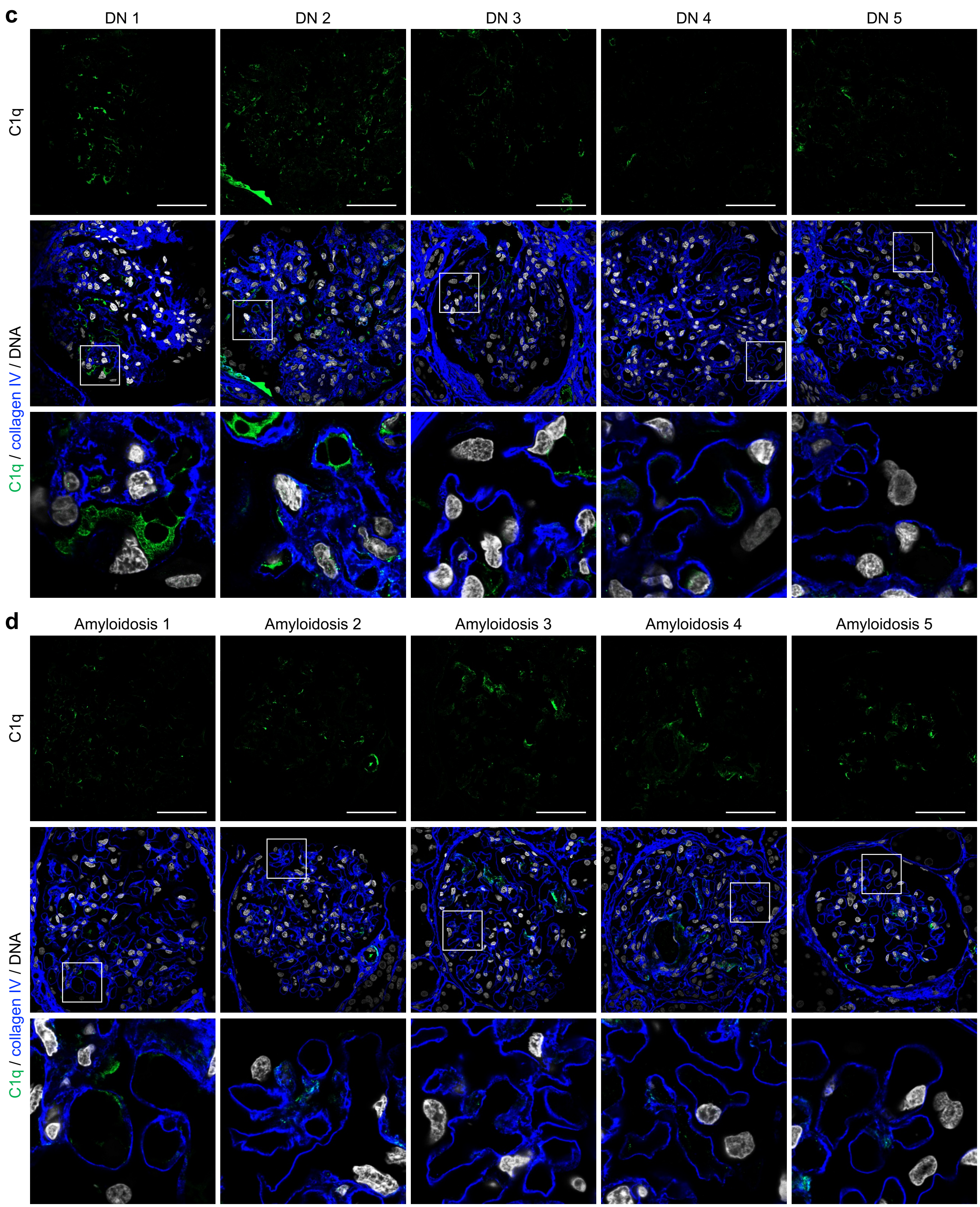

Supplementary Fig. 4 C1q staining in biopsies from patients with different kidney diseases. a-d C1q staining in co-localization with collagen IV in biopsies from patients with primary focal segmental glomerulosclerosis (pFSGS, n=5) (**a**), minimal change disease (MCD, n=5) (**b**), diabetic nephropathy (DN, n=5; cases 1, 3 and 4 are nodular and cases 2 and 5 are diffuse diabetic glomerulosclerosis) (**c**), and amyloidosis (n=5; cases 1-3 are AA amyloidosis, cases 4 and 5 are AL amyloidosis) (**d**). The lower panels represent 5-fold enlargements of the boxed areas in the middle panels. Bars 50 µm.

Supplementary Fig. 5

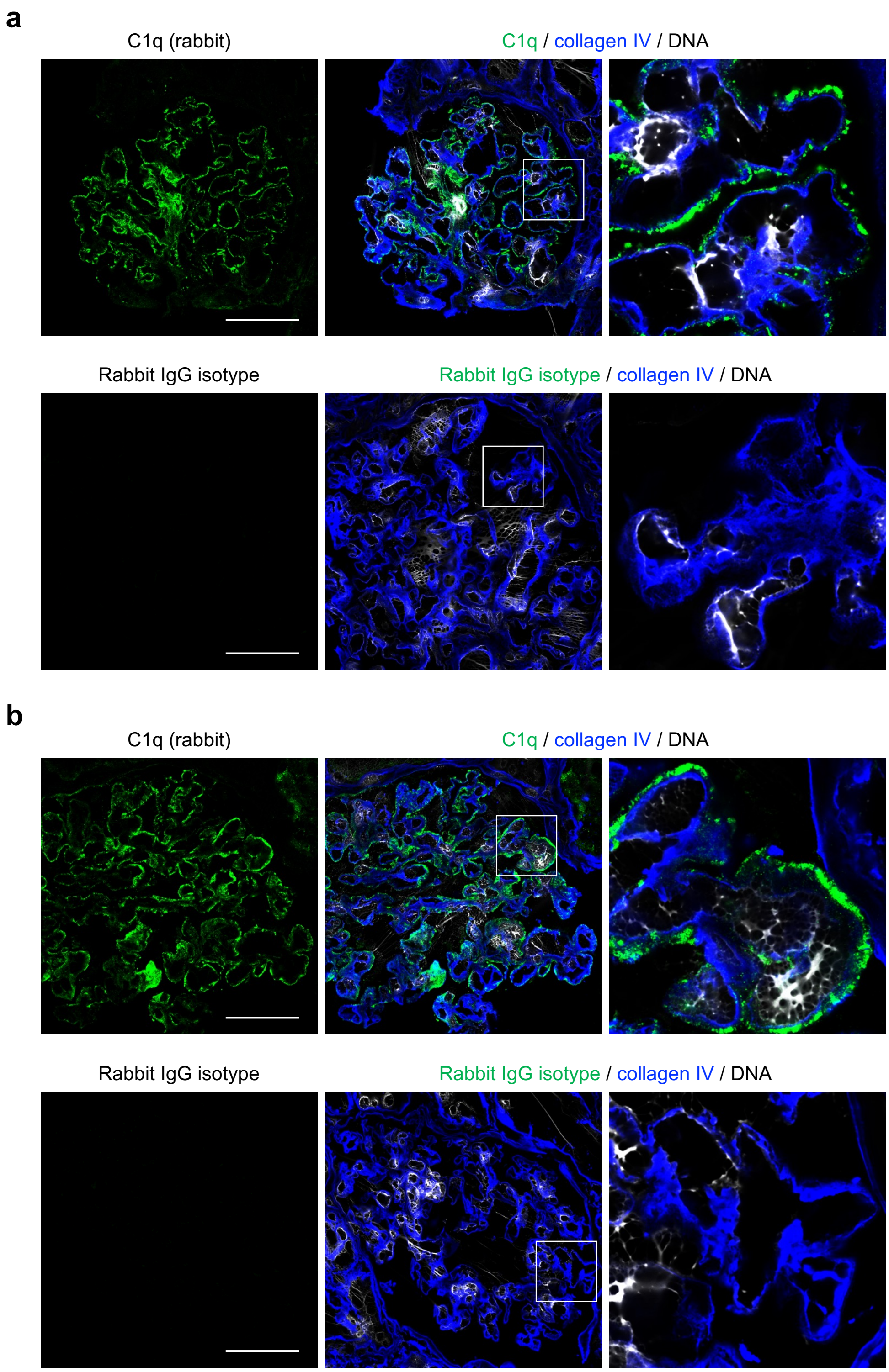

Supplementary Fig. 5 C1q positivity after antigen retrieval in cryoconserved biopsy samples from MN patients is not a consequence of unspecific primary or secondary antibody binding. a, b Representative immunofluorescence stainings for C1q in co-localization with collagen IV in two cases (**a** and **b**) of PLA2R1-associated MN (upper panels). The same staining was performed replacing the primary rabbit anti-C1q antibody with an equally concentrated rabbit IgG isotype control (lower panels). A total of 6 biopsies were investigated like this with congruent results. The right panels represent 5-fold enlargements of the boxed areas in the middle panels. Bars 50 μm.

Supplementary Fig. 6

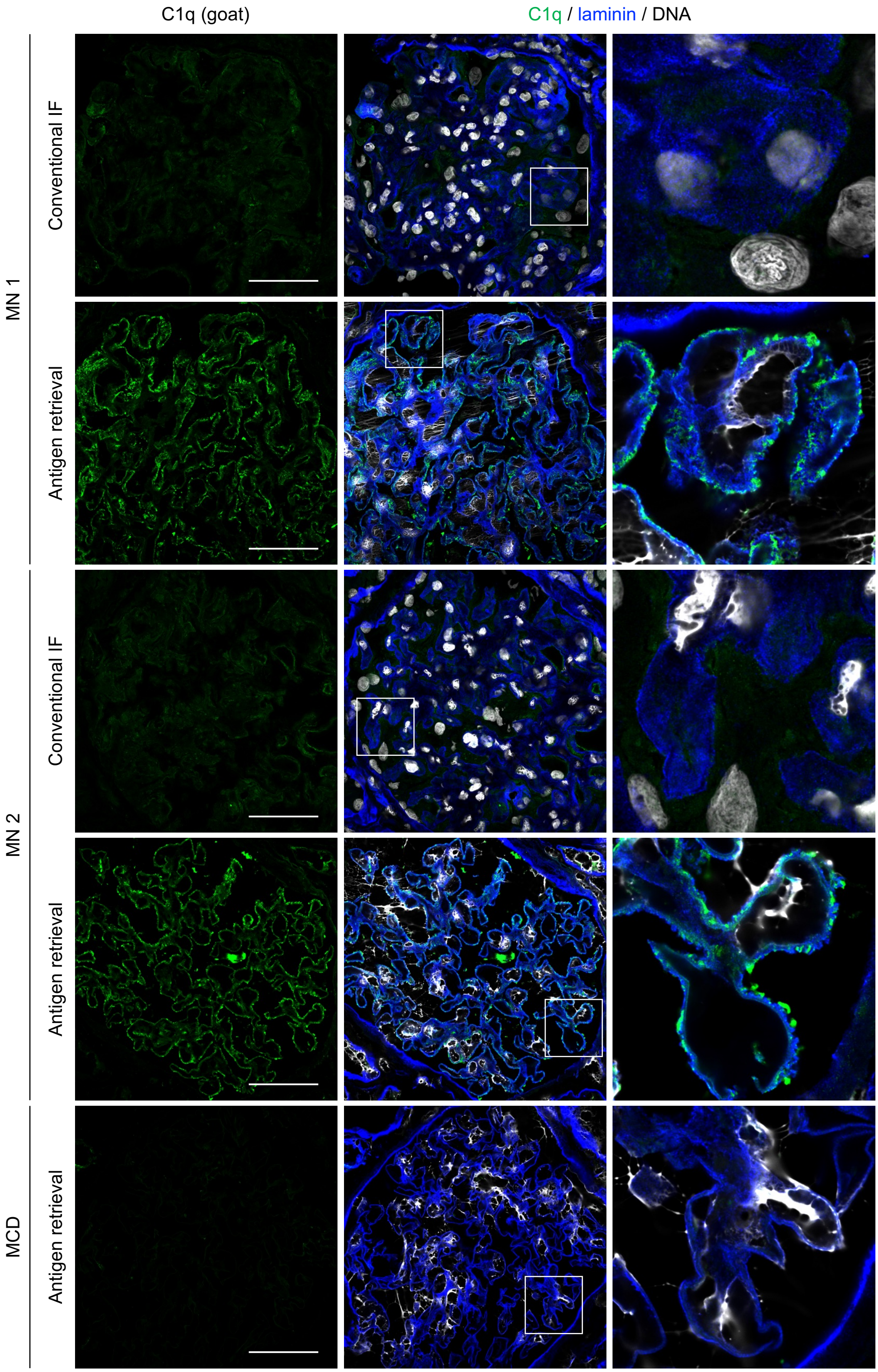

Supplementary Fig. 6 C1q positivity in cryoconserved biopsy samples from MN patients is confirmed with another anti-C1q antibody. Representative immunofluorescence stainings for C1q using a goat anti-C1q antibody in co-localization with laminin in two biopsies from patients with PLA2R1-associated MN (n=6) without and with antigen retrieval and one biopsy from a patient with minimal change disease (MCD) with antigen retrieval (n=2). Panels on the right represent 5-fold enlargements of the boxed areas in the middle panels. Bars 50 µm.

Supplementary Fig. 7

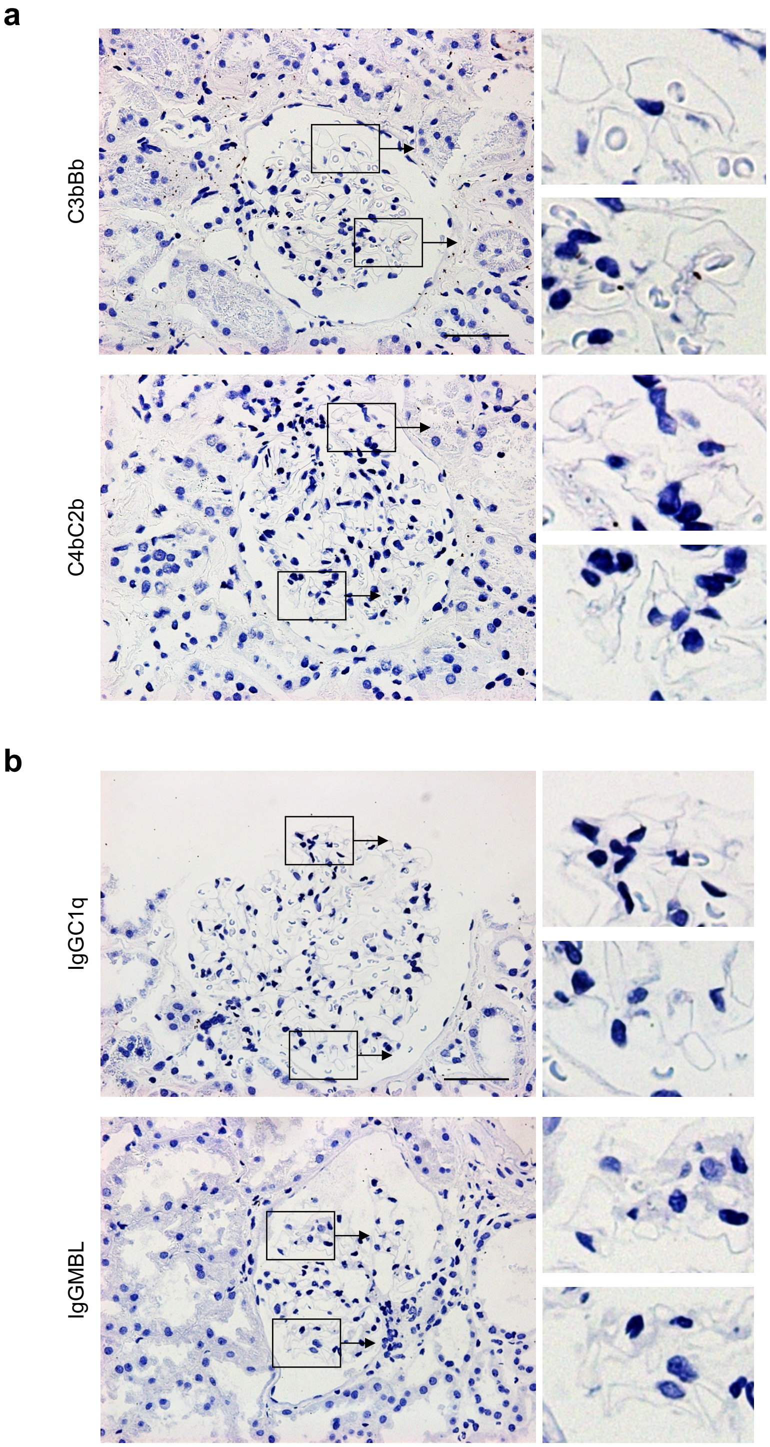

Supplementary Fig. 7 Proximity ligation assay controls. a Representative images of proximity ligation assays for the alternative C3 convertase C3bBb and the classical/lectin C3 convertase C4bC2b performed on time point zero biopsies from renal transplant recipients (n=6). **b** Representative images of proximity ligation assays showing the assemblies of IgGC1q and IgGMBL performed on time point zero biopsies from renal transplant recipients. (n=6). Images on the right are enlargements of the boxed areas in the images on the left. Bars 50 μm.

Supplementary Fig. 8

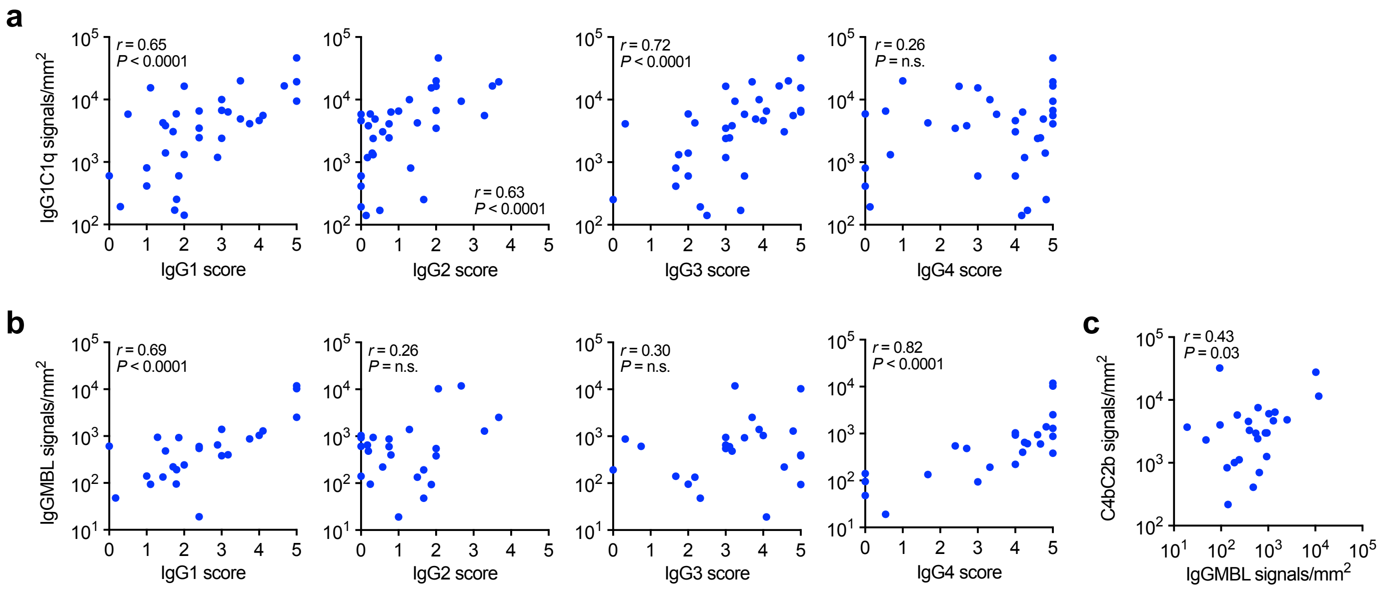

Supplementary Fig. 8 IgG sublcass and proximity ligations assay correlation analyses. a Correlation of the IgG1, IgG2, IgG3 and IgG4 scores with the IgGC1q proximity ligation assay signals. **b** Correlation of the IgG1, IgG2, IgG3 and IgG4 scores with the IgGMBL proximity ligation assay signals. **c** Correlation of the IgGMBL proximity ligation assay signals with the classical/lectin C3 convertase C4bC2b proximity ligation assay signals. Spearman's *r* correlation coefficient (two-tailed).

Supplementary Fig. 9

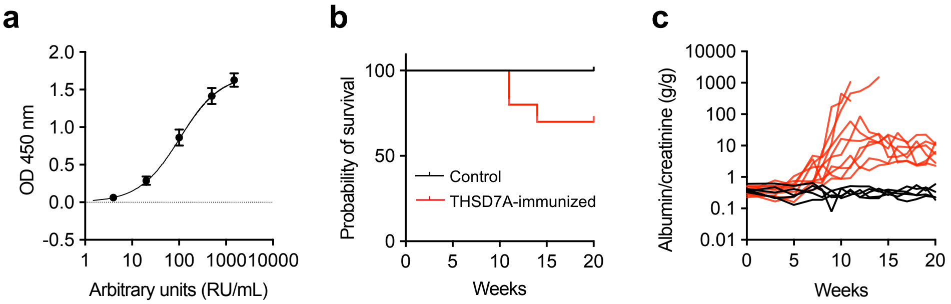

Supplementary Fig. 9 Immunization of mice with THSD7A. **a** ELISA standard curve to determine anti-THSD7A autoantibody titers. Microplates were coated with 100 ng of the extracellular region of murine THSD7A, d1_d21. A mixture of highly positive sera, derived from THSD7A knockout (*Thsd7a*$^{-/-}$) mice that were immunized with murine THSD7A, was used to generate a standard curve consisting of five calibrators (4, 20, 100, 500, and 1500 relative units [RU] per mL). Data are presented as mean and SEM from n=3 independent experiments. **b** Survival analysis. **c** Individual proteinuria over time as measured by albumin-to-creatinine ratio.

Supplementary Fig. 10

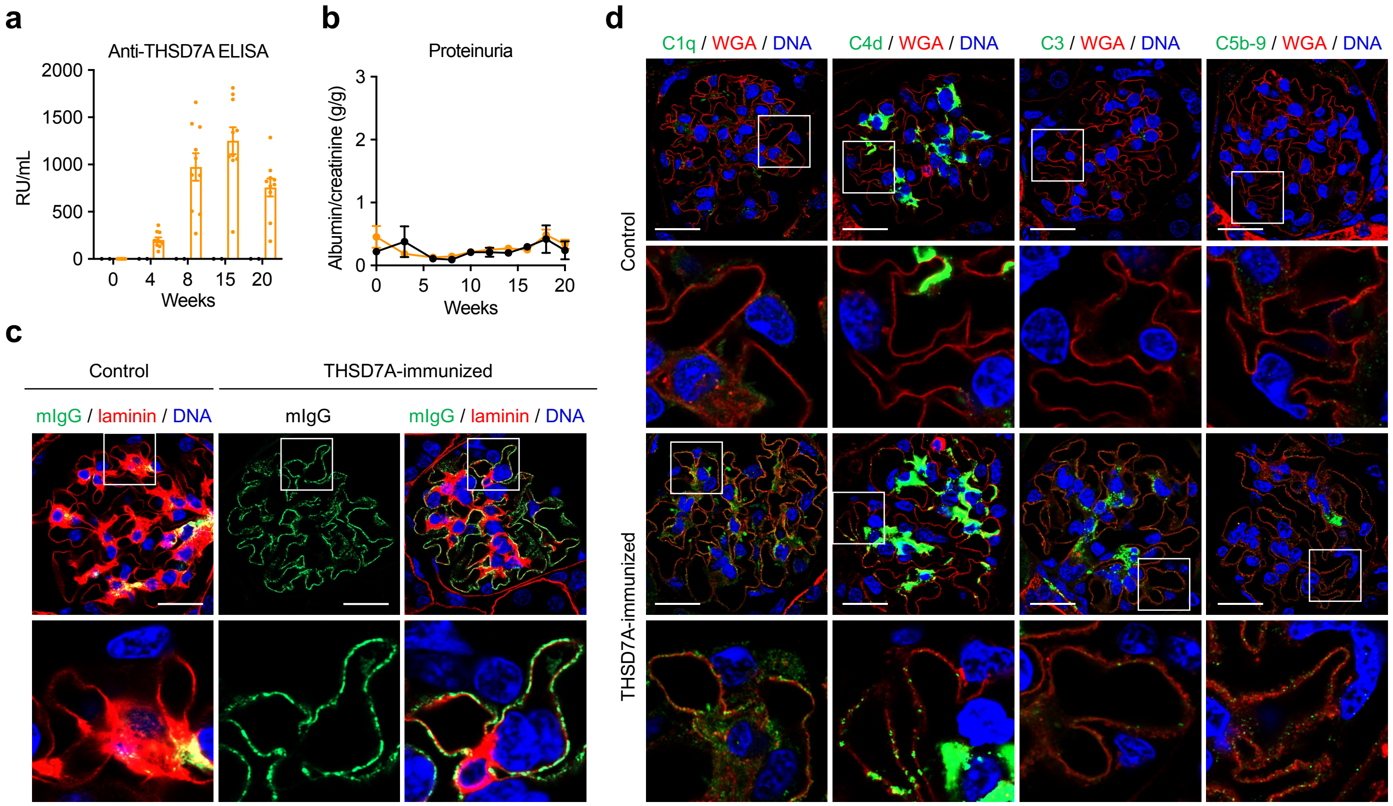

Supplementary Fig. 10 Immunization of C57BL/6 mice. **a, b** Anti-THSD7A antibody titers as measured by ELISA (**a**) and proteinuria as measured by albumin-to-creatinine ratio (**b**) in control (n=2) and THSD7A-immunized (n=10) C57BL/6 mice. Data are presented as mean and SEM. **c** Representative immunofluorescence staining for mouse IgG (mIgG) in co-localization with laminin in control (n=2 analyzed) and THSD7A-immunized (n=5 analyzed) C57BL/6 mice. Bars 20 µm. Lower panels represent 3.5-fold enlargements of the boxed areas in the upper panels. **d** Representative immunofluorescence stainings for complement components C1q, C4d, C3 and C5b-9 in colocalization with wheat germ agglutinin (WGA) in control (n=2) and THSD7A-immunized (n=5) C57BL/6 mice. Bars 20 µm. Lower panels represent 3.5-fold enlargements of the boxed areas in the upper panels.

Supplementary Fig. 11

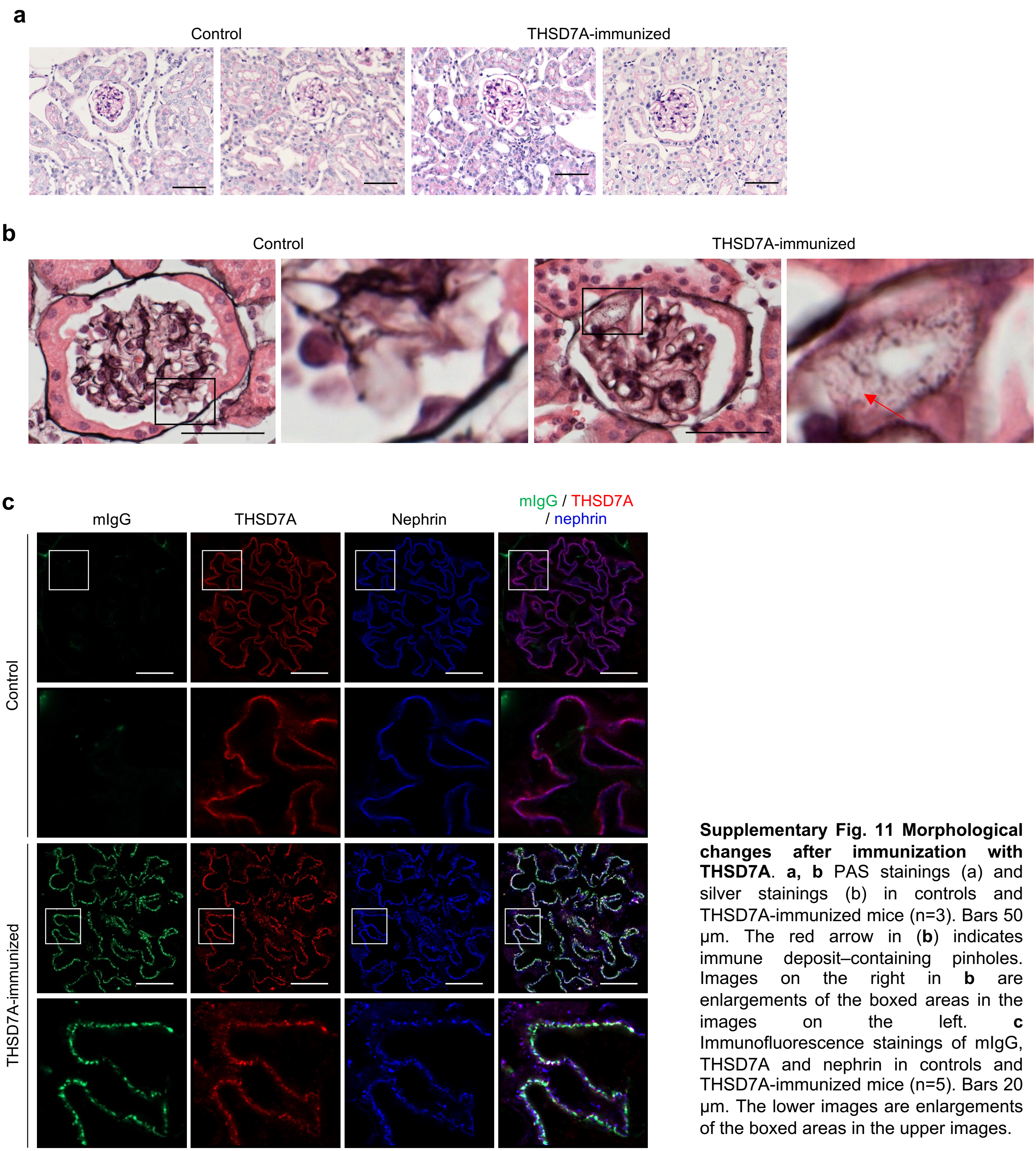

Supplementary Fig. 11 Morphological changes after immunization with THSD7A. **a, b** PAS stainings (a) and silver stainings (b) in controls and THSD7A-immunized mice (n=3). Bars 50 µm. The red arrow in (**b**) indicates immune deposit–containing pinholes. Images on the right in **b** are enlargements of the boxed areas in the images on the left. **c** Immunofluorescence stainings of mIgG, THSD7A and nephrin in controls and THSD7A-immunized mice (n=5). Bars 20 µm. The lower images are enlargements of the boxed areas in the upper images.

Supplementary Fig. 11 *(continued)*

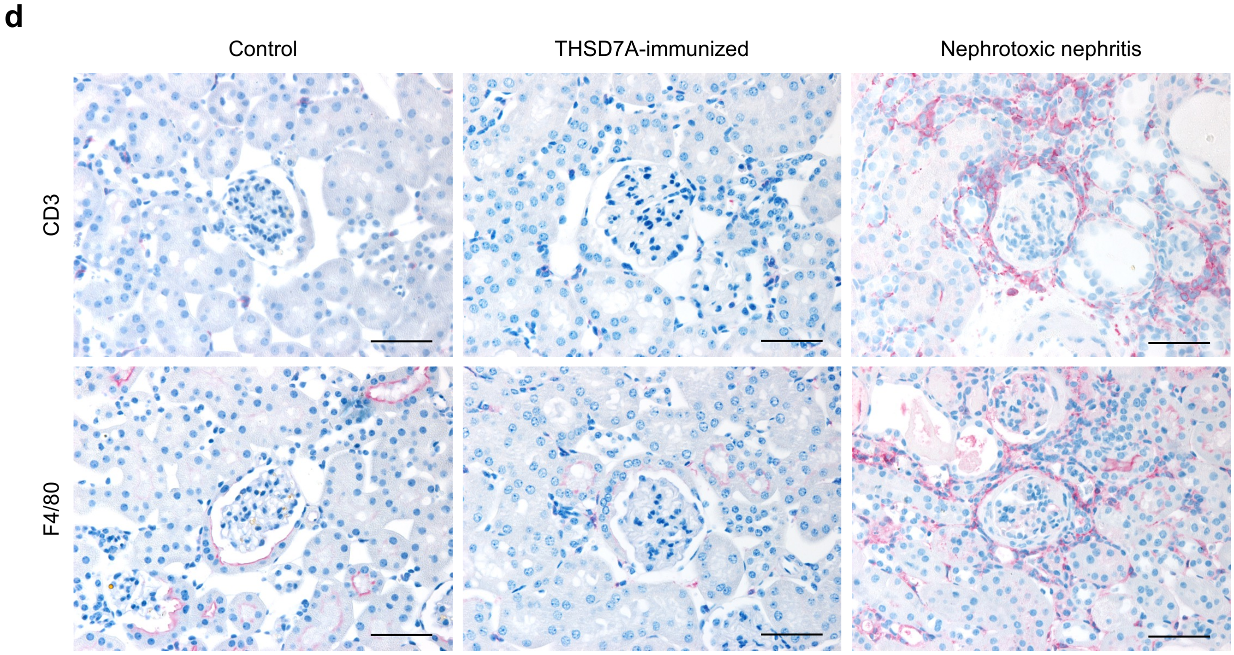

Supplementary Fig. 11 (continued) d Representative images of immunohistochemical stainings for CD3 as a marker of T cells and F4/80 as a marker of macrophages and dendritic cells in control mice and THSD7A-immunized mice (n=2). A sample from a mouse that as induced with the nephrotoxic nephritis model (a model of crescentic glomerulonephritis) was used as a positive control. Bars 50 µm.

Supplementary Fig. 12

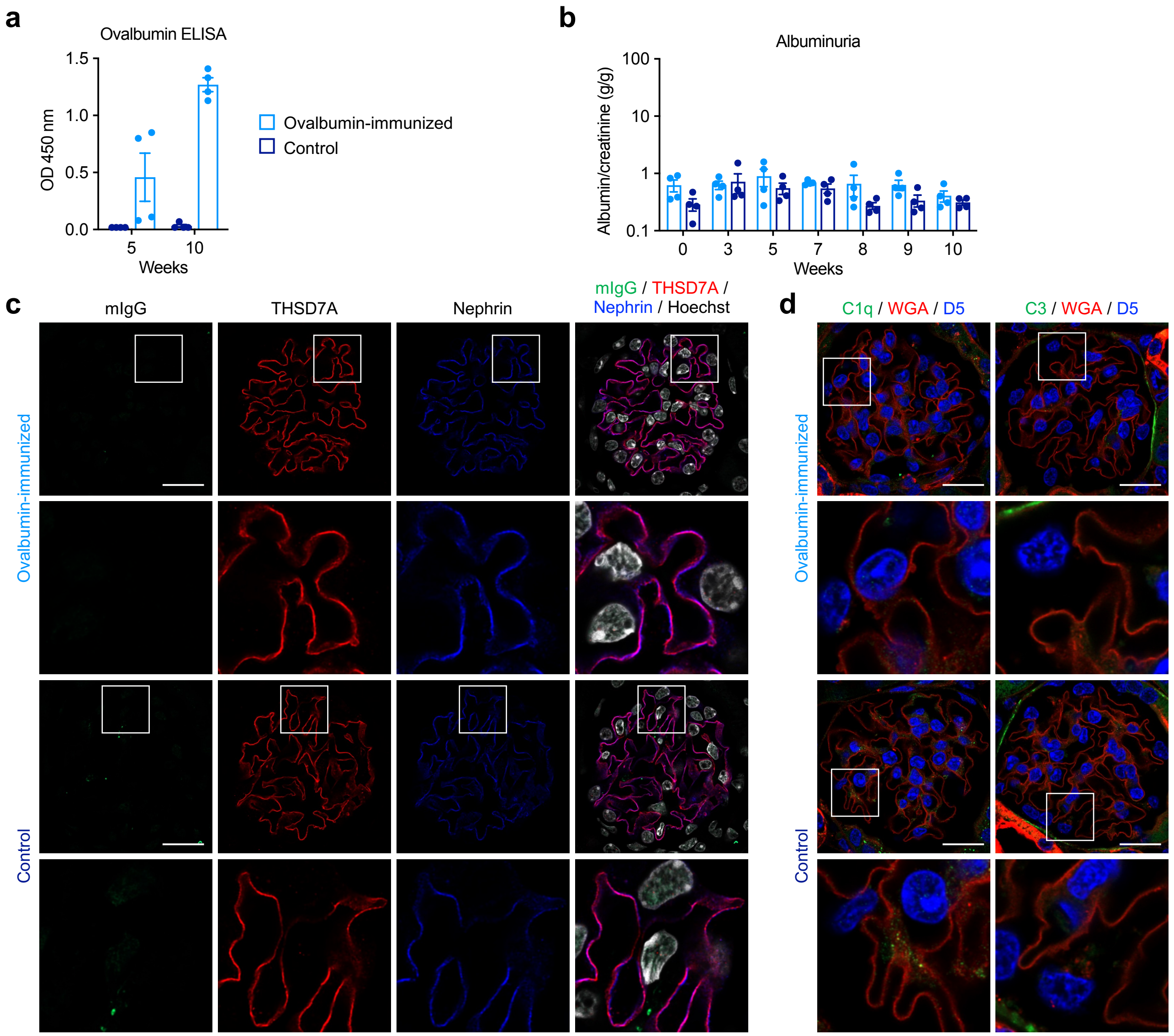

Supplemental Figure 12 Immunization with ovalbumin. a Serum anti-ovalbumin antibody levels as measured by ELISA in ovalbumin-immunized (n=4) and control (n=4) mice. **b** Albuminuria as measured by albumin-to-creatinine ratio in ovalbumin-immunized (n=4) and control (n=4) mice. Data in **a** and **b** are presented as mean and SEM. **c, d** Histological analyses of ovalbumin-immunized mice and controls: representative immunofluorescence stainings for mouse IgG (mIgG) and THSD7A in co-localization with nephrin (**c**), and complement C1q and C3 in co-localization with WGA (**d**) (n=2). Bars 20 µm.

Supplementary Fig. 13

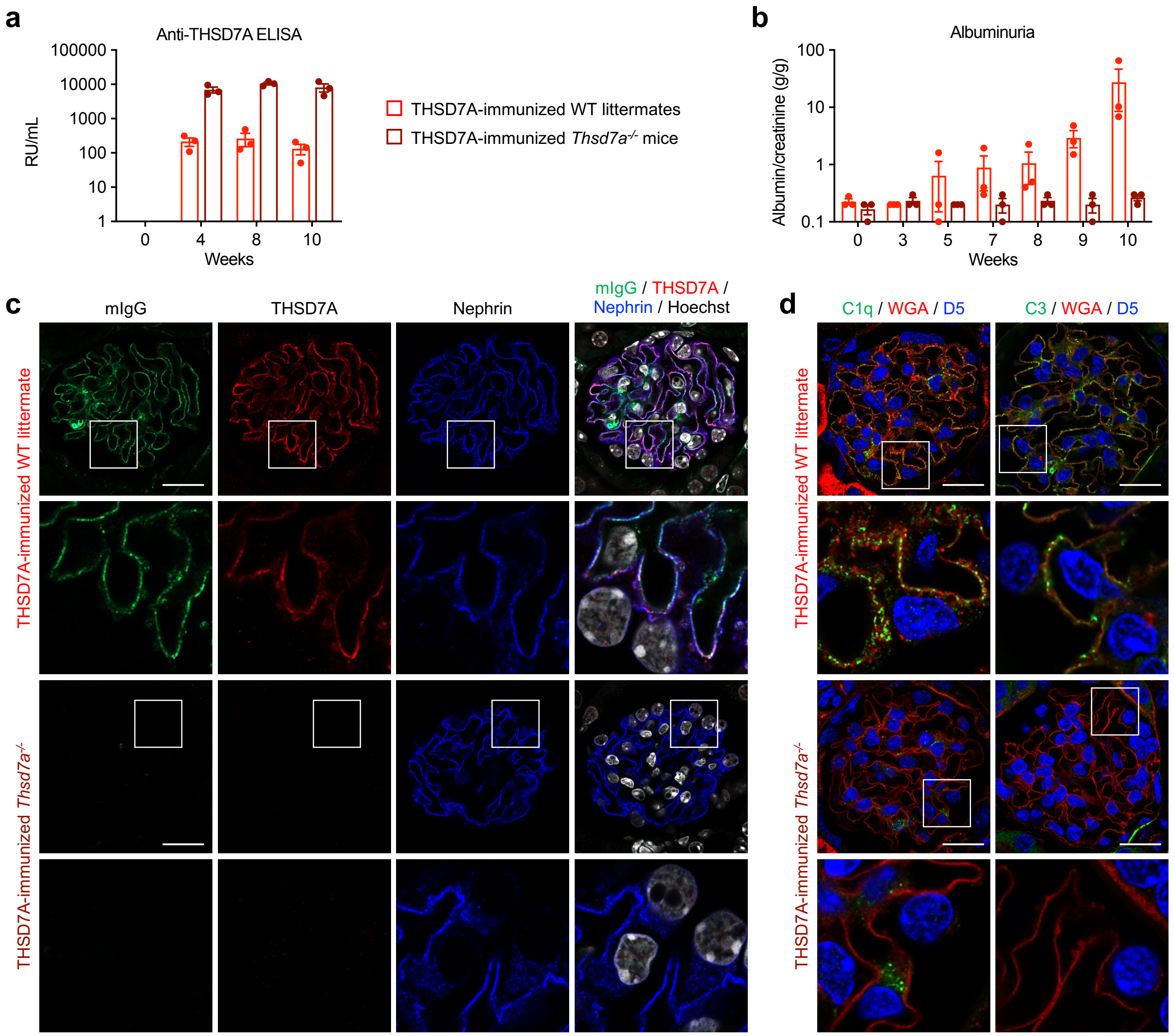

Supplemental Figure 13 Immunization of *Thsd7a*$^{-/-}$ mice and their WT littermates with THSD7A. a Anti-THSD7A serum antibody levels as measured by ELISA in WT littermates (n=3) and *Thsd7a*$^{-/-}$ mice (n=3). **b** Albuminuria as measured by albumin-to-creatinine ratio in immunized WT littermates and *Thsd7a*$^{-/-}$ mice. Data in **a** and **b** are presented as mean and SEM. **c, d** Histological analyses of THSD7A-immunized WT littermates and *Thsd7a*$^{-/-}$ mice: representative immunofluorescence stainings for mouse IgG (mIgG) and THSD7A in co-localization with nephrin (**c**), and complement C1q and C3 in co-localization with WGA (**d**) (n=3). Bars 20 µm.

Supplementary Fig. 14

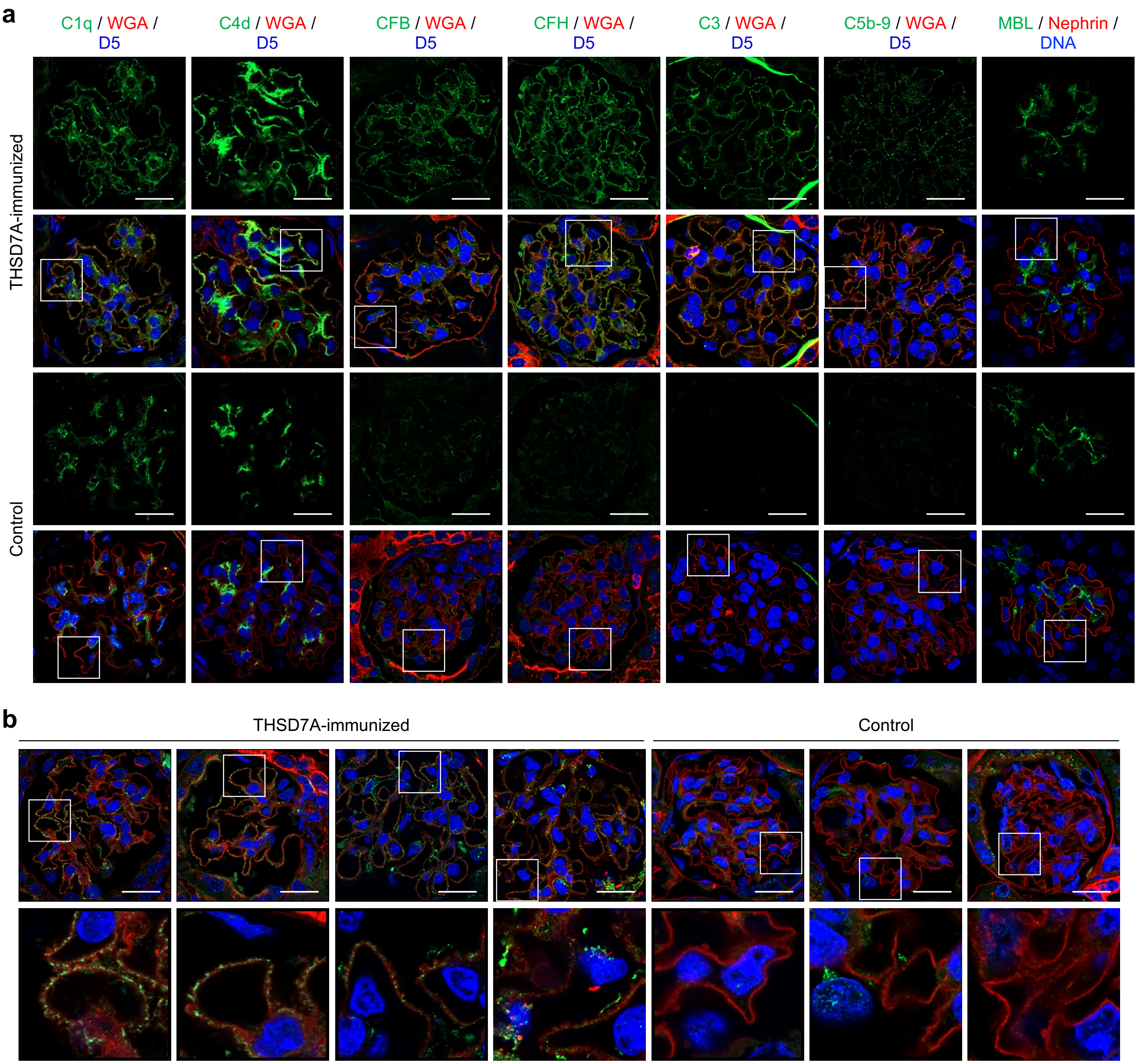

Supplementary Fig. 14 Glomerular complement deposition after immunization with THSD7A a Immunofluorescence stainings of complement C1q, C4d, CFB, CFH, C3 and C5b-9 in colocalization with wheat germ agglutinin (WGA) and MBL in co-localization with nephrin in controls (n=5) and THSD7A-immunized mice (n=10). The boxed areas are shown as enlargements in **Fig. 4e** in the main part. Bars 20 µm. **b** Additional examples of C5b-9 stainings in colocalization with WGA in n=4 THSD7A-immunized and n=3 controls. Bars 20 µm. The lower images are enlargements of the boxed areas in the upper images.

Supplementary Fig. 15

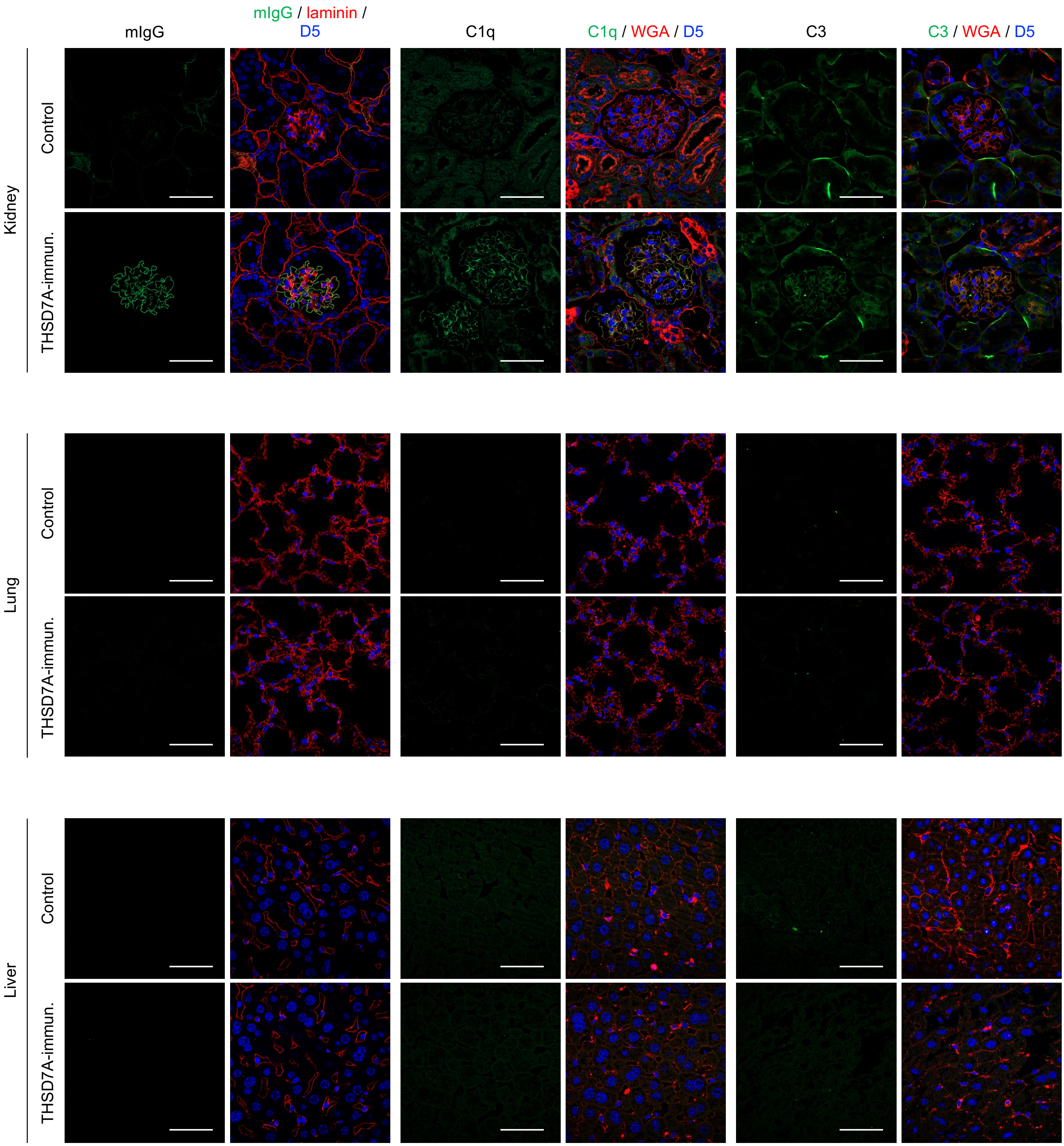

Supplementary Fig. 15 *(continued)*

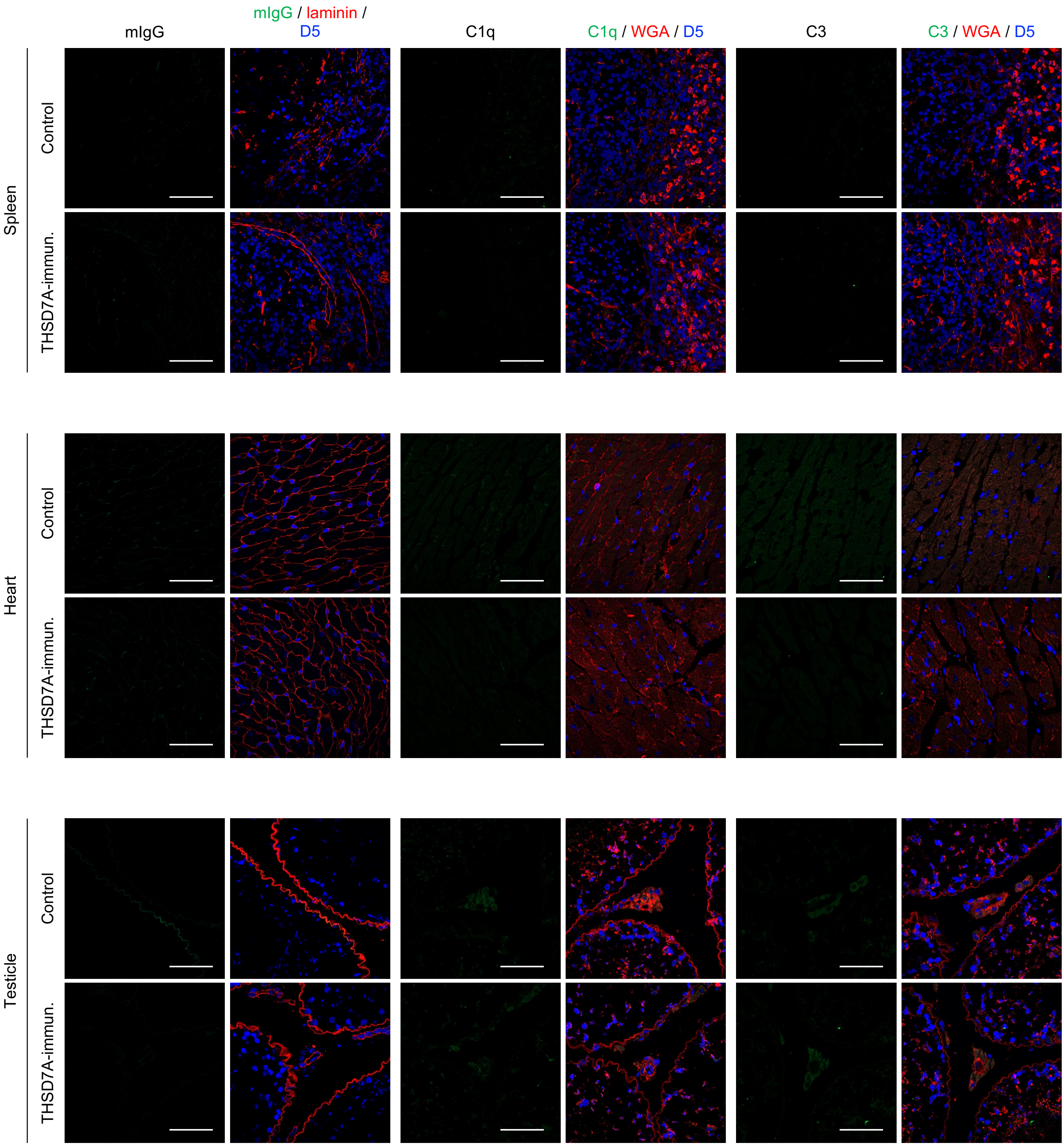

Supplementary Fig. 15 Mouse IgG (mIgG) and complement deposition in various organs. Representative immunofluorescence stainings for mIgG in co-localization with laminin (left), C1q in co-loalization with wheat germ agglutinin (WGA) (middle) and C3 in co-localization with WGA (right) in the kidney, lung, liver, spleen, heart and testes of a control mouse (n=1) and THSD7A-immunized mice (n=2 animals analyzed). Bars 50 µm.

Supplementary Fig. 16

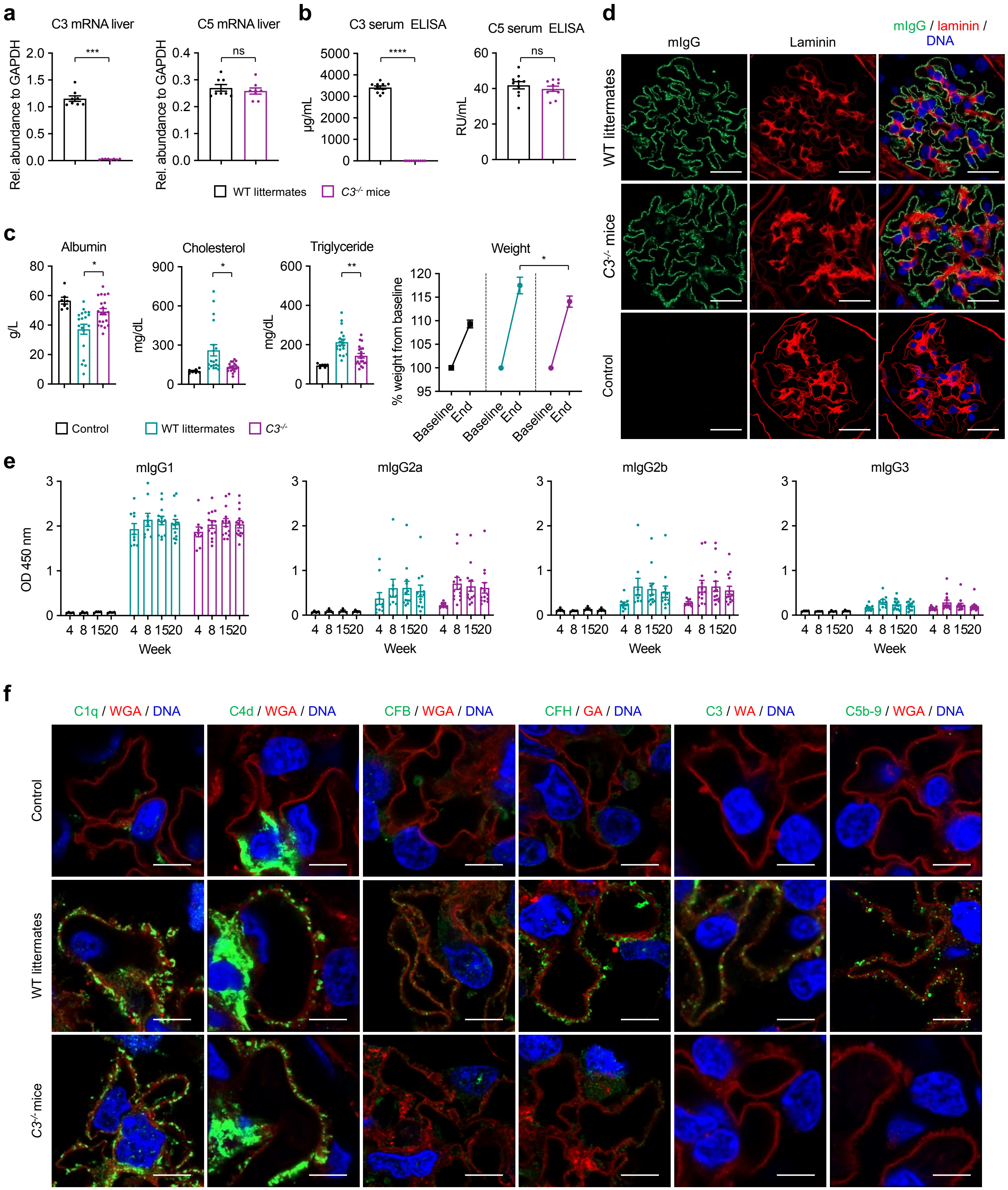

Supplementary Fig. 16 *(continued)*

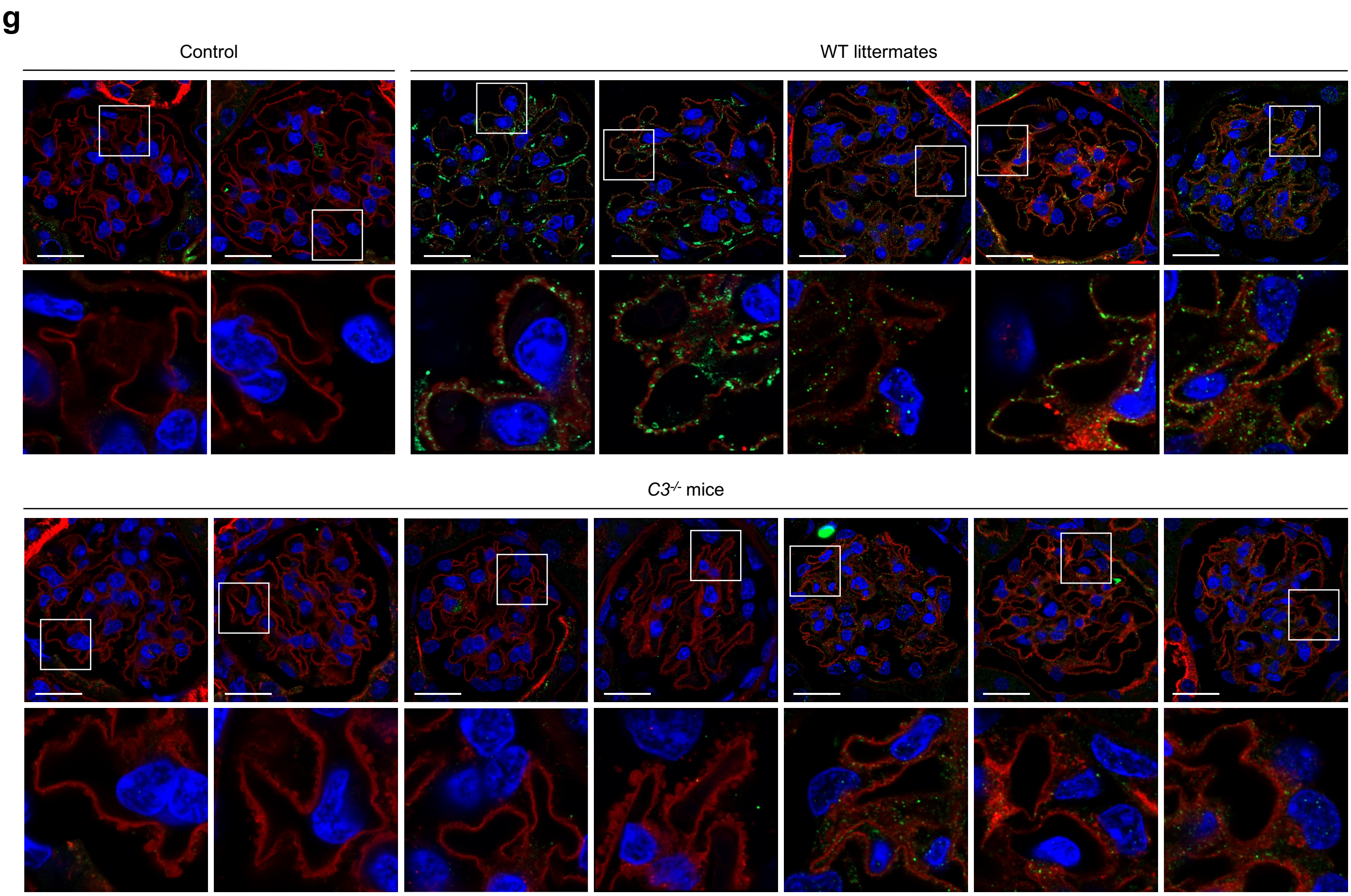

Supplementary Fig. 16 WT littermates and *C3*$^{-/-}$ mice at baseline and after immunization with THSD7A. a C3 (left) and C5 (right) expression in the liver as measured by quantitative PCR in WT littermates and *C3*$^{-/-}$ mice at baseline (n=8). C3, ****p*=0.0002; C5, ns, not significant (two-tailed Mann-Whitney test). **b** Serum levels of complement C3 (left) and C5 (right) as measured by ELISA in WT littermates and *C3*$^{-/-}$ mice at baseline (n=10). *****p*<0.0001; ns, not significant (two-tailed Mann-Whitney test). **c** Serum albumin, cholesterol and triglyceride levels as well as weight change from baseline in control mice (n=7), THSD7A-immunized WT littermates (n=19) and THSD7A-immunized *C3*$^{-/-}$ mice (n=20). Albumin, **p*=0.0495; cholesterol, **p*=0.0267; triglycerids, ***p*=0.0064 (Kruskal-Wallis test with Dunn's correction for multiple comparisons). Data in **a-c** are presented as mean and SEM. **d** Representative immunofluorescence stainings for mIgG in colocalization with the glomerular basement membrane constituent laminin in THSD7A-immunized WT littermates, THSD7A-immunized *C3*$^{-/-}$ mice and control mice (n=3). Bars 20 µm. **e** mIgG subclass-specific anti-THSD7A antibody levels over time as measured by ELISA in control mice (n=4), THSD7A-immunized WT littermates (n=12) and THSD7A-immunized *C3*$^{-/-}$ mice (n=14). Data are presented as mean and SEM. **f** Representative immunofluorescence stainings for complement C1q, C4d, CFB, CFH, C3 and C5b-9 (*n*=6). Bars 4 µm. **g** Additional examples of C5b-9 staining in colocalization with WGA in 2 controls, 5 WT littermates immunized with THSD7A and 7 *C3*$^{-/-}$ mice immunized with THSD7A. Bars 20 µm. The lower images are enlargements of the boxed areas in the upper images.

Supplementary Fig. 17

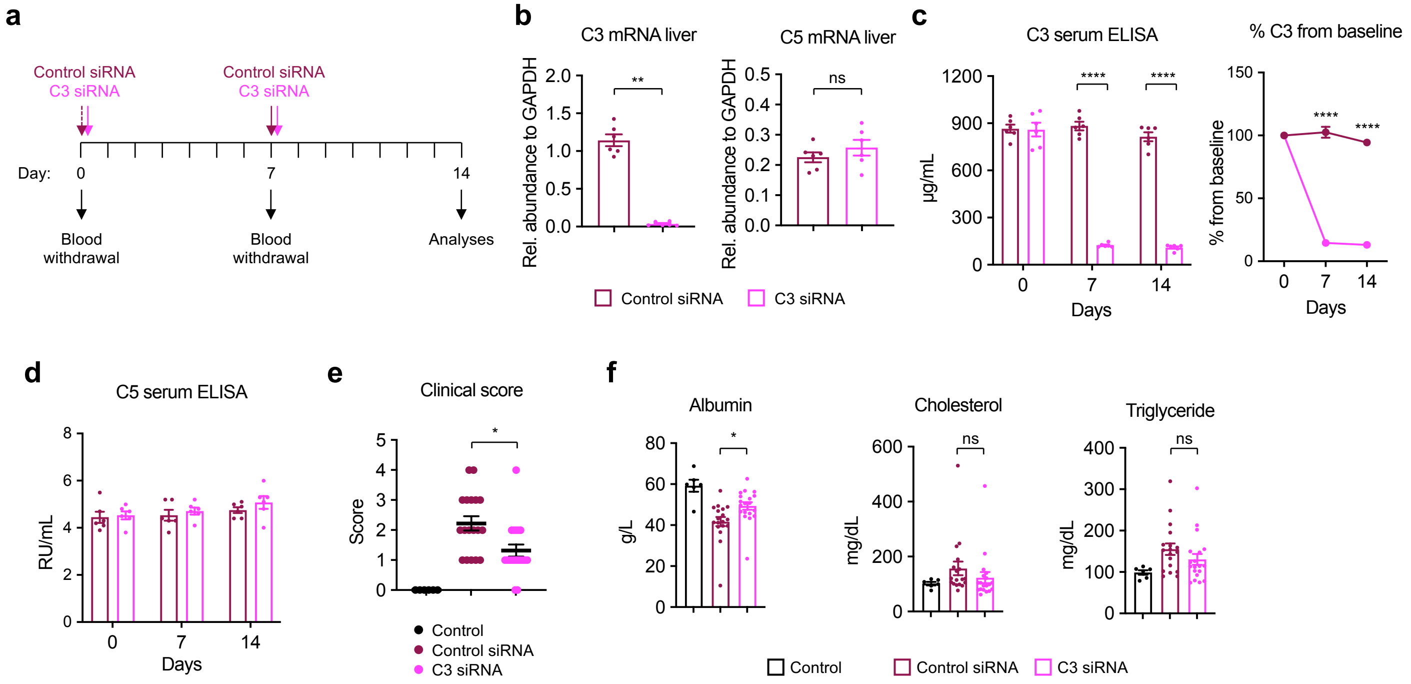

Supplementary Fig. 17 Treatment with C3 siRNA decreases liver and serum C3. **a** Experimental scheme. Mice were injected subcutaneously with C3 siRNA (n=6) or control siRNA (n=6) two times at an interval of seven days. Serum was taken before the first and before the second siRNA application and mice were sacrificed 14 days after the first siRNA application. **b** C3 (left) and C5 (right) expression in the liver as measured by quantitative PCR (n=6). C3, ***p*=0.0022; C5, ns, not significant (two-tailed Mann-Whitney test). **c** Serum levels of complement C3 over time as measured by ELISA (left) and the change in C3 from baseline in percent (right) in animals treated with control siRNA (n=18) and C3 siRNA (n=19). *****p*<0.0001 (two-way ANOVA with Bonferroni correction for multiple comparisons). **d** Serum levels of complement C5 over time as measured by ELISA in animals treated with control siRNA (n=18) and C3 siRNA (n=19). **e** Nephrotic syndrome clinical score. **p*=0.0148 (Kruskal-Wallis test with Dunn‘s correction for multiple comparisons). **f** Serum albumin, cholesterol and triglycerides in control mice (n=6) and THSD7A-immunized mice treated with control siRNA (n=18) or C3 siRNA (n=19). Albumin, **p*=0.0183.; cholesterol and triglycerids, ns, not significant (Kruskal-Wallis test with Dunn‘s correction for multiple comparisons). Data in b-f are presented as mean and SEM.

Membranous nephropathy patients	
Number of patients	39
Number of male/female patients	30/9
Age – years (median)	63
Number of PLA2R1-/THSD7A-positive patients	29/10
Dominant EM stage according to Churgh and Ehrenreich (number)	
I	8
II	17
III	14
IV	0
% IFTA (median)	10

Supplementary Table 1. Clinical characteristics of patients with MN. EM, electron microscopy; IFTA, interstitial fibrosis and tubular atrophy.

Construct	Forward primer	Reverse primer
d1_d2	tcttCCATGGCCGCGGCGCAGGGAGACAC	actaGCGGCCGCCTGGCAAGGGATGAGGC
d10_d11	tcttCCATGGCCGTGTACCACTGGCAAACTGG	actaGCGGCCGCGTGACAAGTGGGAGGGGCTTC
d14_d15	tcttCCATGGCCAAGTACAACGCCCAGCCCGTG	actaGCGGCCGCGTTGCAATCGCTGTGG
d16_d17	tcttCCATGGCCCAGTACCTGTGGGTCACCG	actaGCGGCCGCGTAGCAGTTCTTGTTCAGG
d1_d21	CAGTTCGAAAAATCCGGAATGGCGGCGCAGGGAGACG	GTGGCTCCCTTAGCTCCCTTAGCGGCAGGTCTTCATCTCACT

Supplementary Table 2. Primers for THSD7A mutants.
