# Supplementary material for: The classical pathway triggers pathogenic complement activation in membranous nephropathy
Source: Nat Commun. 2023 Jan 28;14:473. doi: 10.1038/s41467-023-36068-0 (PMC9884226; doi:10.1038/s41467-023-36068-0)
Supplement: Supplementary file 3 — Reporting Summary [file 41467_2023_36068_MOESM3_ESM.pdf]

nature portfolio

Corresponding author(s): Nicola M. Tomas, Thorsten Wiech

Last updated by author(s): Jan 10, 2023

Reporting Summary

Nature Portfolio wishes to improve the reproducibility of the work that we publish. This form provides structure for consistency and transparency in reporting. For further information on Nature Portfolio policies, see our Editorial Policies and the Editorial Policy Checklist.

Statistics

For all statistical analyses, confirm that the following items are present in the figure legend, table legend, main text, or Methods section.

n/a	Confirmed	
[ ]	[x]	The exact sample size (*n*) for each experimental group/condition, given as a discrete number and unit of measurement
[ ]	[x]	A statement on whether measurements were taken from distinct samples or whether the same sample was measured repeatedly
[ ]	[x]	The statistical test(s) used AND whether they are one- or two-sided<br>*Only common tests should be described solely by name; describe more complex techniques in the Methods section.*
[x]	[ ]	A description of all covariates tested
[x]	[ ]	A description of any assumptions or corrections, such as tests of normality and adjustment for multiple comparisons
[ ]	[x]	A full description of the statistical parameters including central tendency (e.g. means) or other basic estimates (e.g. regression coefficient) AND variation (e.g. standard deviation) or associated estimates of uncertainty (e.g. confidence intervals)
[ ]	[x]	For null hypothesis testing, the test statistic (e.g. *F*, *t*, *r*) with confidence intervals, effect sizes, degrees of freedom and *P* value noted<br>*Give P values as exact values whenever suitable.*
[x]	[ ]	For Bayesian analysis, information on the choice of priors and Markov chain Monte Carlo settings
[x]	[ ]	For hierarchical and complex designs, identification of the appropriate level for tests and full reporting of outcomes
[ ]	[x]	Estimates of effect sizes (e.g. Cohen's *d*, Pearson's *r*), indicating how they were calculated

Our web collection on statistics for biologists contains articles on many of the points above.

Software and code

Policy information about availability of computer code

Data collection	No software was used for data collection.
Data analysis	Graph Pad Prism (version 9.4.1), ZEN (blue edition, version 2.6) and ZEN (black edition, version 2.3), Image J (64-bit, version 1.53).

For manuscripts utilizing custom algorithms or software that are central to the research but not yet described in published literature, software must be made available to editors and reviewers. We strongly encourage code deposition in a community repository (e.g. GitHub). See the Nature Portfolio guidelines for submitting code & software for further information.

Data

Policy information about availability of data

All manuscripts must include a data availability statement. This statement should provide the following information, where applicable:

- Accession codes, unique identifiers, or web links for publicly available datasets
- A description of any restrictions on data availability
- For clinical datasets or third party data, please ensure that the statement adheres to our policy

All data supporting the findings of this study are included in the main article, the supplementary information, and the source data that are provided with this manuscript, including fluorescence quantifications and uncropped gels/blots.

Human research participants

Policy information about studies involving human research participants and Sex and Gender in Research.

Reporting on sex and gender	Information on sex was collected for this study. No information on gender was collected.
Population characteristics	MN patients had a median age of 63 years and 77% were male. Characteristics are shown in Supplemental Table 1.
Recruitment	No participants were recruited for this study. Biopsy analyses refer to retrospective analyses of archival tissue.
Ethics oversight	The study of diagnostic human biopsies was conducted in accordance with federal state and institutional guidelines and approved by the local ethics committee of the Chamber of Physicians in Hamburg (registration number PV5541).

Note that full information on the approval of the study protocol must also be provided in the manuscript.

Field-specific reporting

Please select the one below that is the best fit for your research. If you are not sure, read the appropriate sections before making your selection.

☒ Life sciences ☐ Behavioural & social sciences ☐ Ecological, evolutionary & environmental sciences

For a reference copy of the document with all sections, see nature.com/documents/nr-reporting-summary-flat.pdf

Life sciences study design

All studies must disclose on these points even when the disclosure is negative.

Sample size	Sample size was determined based on the experience with the model and complexity of the experiments.<br>The calculation of the required number of animals is derived from the respective expected biologically relevant difference of the mean values of the two experimental groups and the expected standard deviation, which was estimated on the basis of the development of the model experimental autoimmune membranous nephropathy. A power of 80% with a type 1 error probability of <5% was required for group size calculations.<br>No sample size calculation was performed for the analysis of human biopsy tissue. The sample size of n=39 human biopsies from patients with MN was chosen on availability of sufficient material for the analyses. The number of n=39 can be considered sufficient to get a representative dataset for the distribution of the four IgG subclasses as well as the herein investigated four complement component assemblies in biopsies from patients with primary membranous nephropathy.
Data exclusions	No data were excluded.
Replication	The number of replications for each experiment is indicated in the Figure legend and/or main text.
Randomization	Mice were randomized to the groups. No randomization was done for the analyses of human biopsy material as this involved no intervention.
Blinding	The investigators were blinded during the generation of the data (stainings, measurement of clinical parameters). Analyses of quantitative data was not conducted in a blinded manner as measurements were not affected by the experimentator. If quantifications were performed from histological images, stainings, pictures and scoring were performed in a blinded manner by three different investigators. Mice were allocated to experimental groups in a blinded manner. Histological analyses were done blinded to genotype (C3 knockout versus wild-type) and treatment (siRNA).

Reporting for specific materials, systems and methods

We require information from authors about some types of materials, experimental systems and methods used in many studies. Here, indicate whether each material, system or method listed is relevant to your study. If you are not sure if a list item applies to your research, read the appropriate section before selecting a response.

Materials & experimental systems

n/a	Involved in the study
☐	☒ Antibodies
☐	☒ Eukaryotic cell lines
☒	☐ Palaeontology and archaeology
☐	☒ Animals and other organisms
☒	☐ Clinical data
☒	☐ Dual use research of concern

Methods

n/a	Involved in the study
☒	☐ ChIP-seq
☒	☐ Flow cytometry
☒	☐ MRI-based neuroimaging

Antibodies

Antibodies used

For immunolocalization in human biopsy samples, the following antibodies were used: collagen IV (goat, 1:400, Southern Biotech 1340-01), human IgG (Cy2 or Cy3 donkey anti-human IgG, 1:200; Jackson ImmunoResearch Laboratories), C2 (mouse, 1:10; Santa Cruz, sc373809), C4b (rabbit, 1:500; Abcam ab181241), CFB (rabbit, 1:20; Proteintech 10170-1-AP), C3b (mouse, 1:1000; Abcam ab11871), C1q (rabbit, 1:1500; Dako A0136), C1q (goat serum, 1:400; Complement Technology A200), MBL (rabbit, 1:800; Abcam ab190834). For the localization of human IgG subclasses, monoclonal antibodies against IgG1 (mouse, 1:100, SouthernBiotech, 9052-01, clone no. 4E3), IgG2 (mouse, 1:40000, SouthernBiotech, 9080-01, clone no. HP6014), IgG3 (mouse, 1:1000, SouthernBiotech, 9210-01, clone no. HP6050), and IgG4 (mouse, 1:4000, SouthernBiotech, 9200-09, clone no. HP6025) were used.
For immunolocalization in mice, the following antibodies were used: nephrin (guinea pig, 1:200; Acris Antibodies BP5030), neph1 (guinea pig, 1:200; kindly provided by Florian Grahammer), laminin (rabbit, 1:1000; Sigma-Aldrich L9393), WGA-rhodamin (1:400; Vector-Laboratories), THSD7A (goat, 1:200; Santa Cruz Biotechnology sc163455 or rabbit, 1:200; Sigma-Aldrich HPA000923), C1q (goat serum, 1:400; Complement Technology A200), C3 (FITC goat anti-C3, 1:100; Cappel 55500), C4d (rabbit, 1:50; Hycultec HP8033), C5b-9 (rabbit, 1:200; Abcam ab55811), CFB (goat, 1:50; Complement Technology A235), CFH (goat, 1:100; Complement Technology A237), MBL (rabbit, 1:800; Abcam ab190834), murine IgG (Cy2 donkey anti-mouse IgG H+L, 1:200; Jackson ImmunoResearch Laboratories), murine IgG subclasses (IgG1, IgG2a, IgG2b, IgG3) (goat, 1:5000; Rockland 610-101-040, 610-101-041, 610-101-042, 610-101-043, respectively), synaptopodin (guinea pig, 1:200; Synaptic Systems 163004 or rabbit, 1:400; Santa Cruz 50459), DACH-1 (rabbit, 1:100; Sigma-Aldrich HPA012672), 8-oxoguanine (goat, 1:600; Abcam ab10802).
The following flurochrome-conjugated secondary antibodies were used (all 1:200, Jackson ImmunoResearch Laboratories): anti-goat IgG AF488 (705-545-147), anti-goat IgG Cy3 (705-165-147), anti-guinea pig IgG Cy5 (706-175-148), anti-mouse IgG Cy2 (715-225-150), anti-mouse IgG Cy3 (715-165-150), anti-rabbit IgG AF488 (711-545-152), anti-rabbit IgG Cy3 (711-165-152).

Validation

All antibodies against human proteins are routinely used in the Department of Pathology. During establishment of a staining, antibodies are routinely tested in comparison with an isotype control in the same concentration followed by the same secondary antibody.
All antibodies against mouse proteins are routinely used in the lab (PMIDs: 27214550, 28814510, 32033781, 36191868).

Eukaryotic cell lines

Policy information about cell lines and Sex and Gender in Research

Cell line source(s)

HEK293T cells derived from the DSMZ-German Collection of Microorganisms and Cell Cultures GmbH, product number ACC 635. HEK293-6E cells were kindly provided by Yves Durocher, Ottawa, Canada (PMID: 33827946).

Authentication

The cell lines were not authenticated.

Mycoplasma contamination

Cell lines were regularly tested for mycoplasma contamination. Only negative tested cells were used.

Commonly misidentified lines
(See ICLAC register)

No commonly misidentified cell lines were used in the study.

Animals and other research organisms

Policy information about studies involving animals; ARRIVE guidelines recommended for reporting animal research, and Sex and Gender in Research

Laboratory animals

The study involved wildtype BALB/c mice, wildtype C57BL/6 mice as well as C3-deficient mice (C3 knockout mice: B6:129S4-C3tmCrr/J (stock no. 003641), purchased from The Jackson Laboratory (Farmington, CT, USA)) on the BALB/c background. Thsd7a knockout first mice (Thsd7atm1a mice) were obtained from the Mutant Mouse Resource & Research Centers, MMRRC, stock no. 063558-UCD). All mice were immunized at the age of 12 weeks and then monitored for a maximum of 20 weeks, i.e. until the age of 32 weeks. Mice were housed in a specific-pathogen-free facility at temperatures of 21–24°C with 40–70% humidity on a 12h light/12h dark cycle and provided with food and water ad libitum.

Wild animals

No wild animals were used in this study.

Reporting on sex

Only male mice were used in animal experiments.

Field-collected samples

No field-collected samples were used in this study.

Ethics oversight	Animal experiments were performed according to national and institutional animal care and ethical guidelines and were approved by the Veterinarian Agency of Hamburg and the local animal care committee (registration numbers 114/18 and 002/19).

Note that full information on the approval of the study protocol must also be provided in the manuscript.
